# Effect analysis of neural network robot system in music relaxation training to alleviate adverse reactions of chemotherapy in patients with breast cancer

**DOI:** 10.3389/fnbot.2023.1120560

**Published:** 2023-01-19

**Authors:** Yue Teng, Jinlei Bao, Yinfeng Li, Haichun Ye

**Affiliations:** ^1^College of Human and Health Sciences, Swansea University, Swansea, United Kingdom; ^2^College of Nursing, Shandong Xiehe University, Jinan, Shandong, China; ^3^School of Medicine, Sichuan Cancer Hospital and Institute, Sichuan Cancer Centre, University of Electronic Science and Technology of China, Chengdu, China; ^4^Department of Geriatric Services and Management, School of Humanities Education, Ningxia Vocational Technical College of Industry and Commerce, Yinchuan, China

**Keywords:** breast cancer patients, alleviate adverse reactions, music therapy, neural network robot system, breast cancer

## Abstract

Music therapy is a common method to relieve anxiety and pain in cancer patients after surgery in recent years, but due to the lack of technical and algorithmic support, this therapy is not particularly stable and the therapeutic effect is not good. In this study, a neural network robotic system based on breast cancer patients was designed to analyze the effect of music relaxation training on alleviating adverse reactions after chemotherapy in breast cancer patients. Firstly, this paper introduces the necessity of neural network robot system research under the background of music therapy, and then summarizes the positive effect of music relaxation therapy on alleviating adverse reactions after chemotherapy in breast cancer patients, finally, uses neural network robot system to construct music therapy system. The experimental results show that the new music therapy proposed in this study has a good effect in alleviating the adverse reactions of breast cancer patients after chemotherapy, and the cure rate is increased by 7.84%. The research results of this paper provide reference for the next development of neural network robot system in the medical field.

## 1. Introduction

Breast cancer patients have to give up or interrupt chemotherapy because they cannot tolerate the adverse reactions brought by chemotherapy, which ultimately affects the therapeutic effect. The purpose of this manuscript is to use music relaxation training to alleviate adverse reactions during chemotherapy, promote muscle and nerve relaxation of patients, reduce anxiety and pain of patients, and provide reference value for improving the quality of life of patients and formulating cure measures.

Alleviating the adverse reactions of patients during treatment is the focus of treatment work, and also the research focus of the medical community. Fu Yali evaluated the adverse effects of multi-target tyrosine kinase inhibitors in the treatment of gastrointestinal tumors. Finally, it was concluded that multi target tyrosine kinase inhibitors have a good effect in the treatment of mild or moderate adverse reactions (Fu et al., 2018). Ji Jing analyzed the effect of high-quality nursing on relieving adverse reactions of liver cancer treatment. Finally, high-quality nursing intervention in the treatment of liver cancer can reduce the incidence of surgical pain and postoperative adverse reactions (Jing, 2020). Deshmukh Vineeta applied the nano particle system to the adverse reactions during cancer chemotherapy. Practice showed that this system cannot only explore the delivery effect of drugs, but also reduce the systemic toxicity of patients (Vineeta, 2021). Yang Qian explored the role of chemical photothermal combination therapy in inhibiting adverse reactions of cancer chemotherapy by constructing a porous nano particle system. The research showed that this therapeutic approach showed a strong anti-cancer therapeutic effect (Qian et al., 2018). Giavina-Bianchi Pedro reviewed the common adverse reactions caused by cancer chemotherapy drugs - hypersensitivity reactions. It was concluded that rapid drug desensitization can minimize allergic reaction when hypersensitivity occurs (Giavina-Bianchi et al., 2017). Demaria Marco believed that many genotoxic chemotherapy would produce adverse reactions, and pointed out that aging cells would cause some side effects of chemotherapy, and provide a new target for reducing the toxicity of anti-cancer therapy (Marco, 2017). Singh Kanchanlata discussed the effects of different antioxidants and their analogues on adverse reactions during chemotherapy. Comprehensive data showed that antioxidant supplementation during chemotherapy can improve the therapeutic effect and improve the quality of life of patients (Singh et al., 2018). These researches on relieving adverse reactions of patients are still of reference value, but they have not been applied to music relaxation training.

With the continuous updating of therapeutic techniques, music relaxation training (music therapy) has achieved good results in recent years. Sandler Hubertus analyzed the role of compact disc music in the treatment of patients with depression or anxiety disorder, and finally found that some excellent music can induce patients to have a relaxed state and subjective well-being (Hubertus, 2017). Ghezeljeh T. Najafi studied the effects of massage and music on pain intensity and anxiety intensity of burn patients. The research results showed that music, massage and the combination of these two interventions are effective in reducing pain and anxiety intensity (Najafi Ghezeljeh et al., 2017). Nelson Kirsten analyzed the effect of introducing music to adolescents before surgery to reduce pain and anxiety. Practice showed that during the operation, the pain and anxiety of teenagers have been significantly reduced (Nelson et al., 2017). Liao Juan summarized the current situation of the application of the five elements music therapy in the depression psychology of cancer patients. The study found that, as a simple and effective intervention, this treatment method reduced the anxiety and depression of cancer patients, and provided an effective way for better self-management in cancer treatment (Liao et al., 2018). Bradt Joke applied music relaxation training to dental treatment, and finally found that the anti-anxiety effect of music can reduce the anxiety state and pain of patients to a certain extent (Bradt and Teague, 2018). Pradopo Seno combined aromatherapy and music therapy for dental treatment of pediatric patients. Practice showed that aromatherapy and music therapy can divert children’s attention and reduce their anxiety (Pradop et al., 2017). Golino Amanda J studied the effect of active music therapy intervention on physiological parameters of patients in intensive care unit. The study found that the sleep quality of patients was significantly improved after music intervention (Golino et al., 2019). The above application of music therapy in the medical field is more detailed, but it does not involve the alleviation of adverse reactions of chemotherapy in breast cancer patients.

During the treatment of breast cancer, many patients are troubled by the adverse reactions caused by chemotherapy all the time, and what people can do now is to alleviate these adverse reactions. Conventional treatment methods have certain defects, and it is inevitable that patients’ psychological and physiological problems would not be taken into account. Music relaxation training is used to alleviate adverse reactions of patients, which is conducive to reducing anxiety and pain. In this manuscript, neural network robot system is applied to music therapy to improve the relief effect of music relaxation training and change the postoperative quality of life of patients. Experimental results show that the music therapy method proposed in this manuscript has a good effect in alleviating the adverse reactions of breast cancer patients during chemotherapy, reducing the anxiety and pain of patients.

## 2. Music therapy in the context of neural network robot system

In recent years, robot system has become an important research and application field. Many robots are used to complete complex and even dangerous tasks. With the progress of computer science and technology such as sensor technology, medical imaging technology, artificial neural network technology, and modern information processing, robot technology has been applied to the medical field, resulting in medical robots. Medical robots are mainly used for analyzing patients’ conditions, assisting in surgery, therapeutic training, rehabilitation treatment, etc. Because medical data needs to be stored in large quantities, medical robots must perform a large amount of calculations to complete complex tasks, and neural networks can calculate and integrate large amounts of data, which is why neural network robot systems are gradually entering the medical field.

**FIGURE 1:**
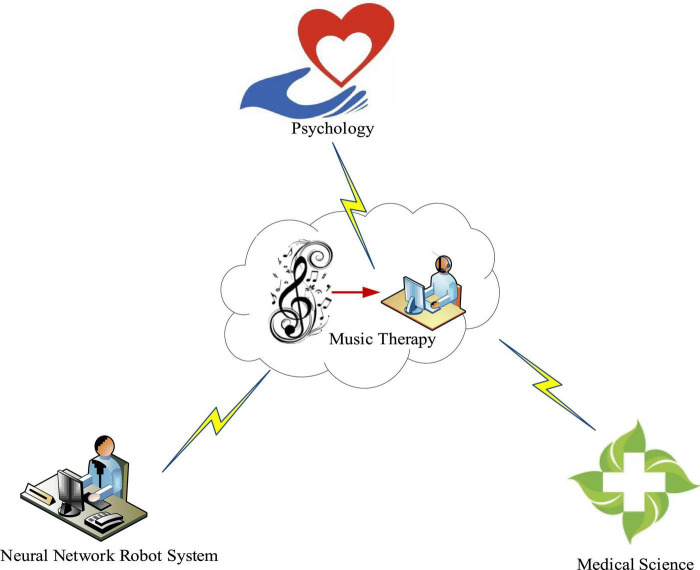
Scope of music therapy.

With the increasing emphasis on music therapy in the medical community, its development space has been greatly improved. As shown in Figure 1, music therapy is a relatively complex discipline, which includes not only medicine, but also psychology and neural network robot system. AI is a very broad science, which consists of different fields, such as machine learning, computer vision, etc. In general, one of the main goals of AI research is to enable machines to be competent for some complex tasks that usually require human intelligence. Under the background of neural network robot system, music therapy is used for various diseases, such as mental disease, nervous system, cardiovascular disease, etc. As a new therapeutic approach, music therapy is not mature enough, and problems such as untimely monitoring of the treatment process and difficult evaluation of the treatment effect often occur. The emergence of neural network robot system has brought a development opportunity for the innovation of treatment methods. The application of neural network and robot technology to music therapy can further improve the monitoring effect and the accuracy of information acquisition. In addition, relevant evaluation models and music recommendation systems can also be established by using relevant machine learning algorithms.

## 3. Necessity of music relaxation training

Relaxation training can offset the negative effects of physiological and psychological stress, restore the balance and coordination of human body, psychology and spirit, help individuals to face the challenges of life in a healthier way, and make the human body’s involuntary reactions, such as heartbeat, respiration, blood pressure, and adrenaline secretion under autonomous control. At present, relaxation training has been widely used in patients with bronchial asthma, coronary heart disease, hypertension, diabetes, cancer, surgery, childbirth and chemotherapy, and has achieved relatively positive results. During chemotherapy for cancer patients, relaxation training can improve their anxiety, depression and other negative emotions by reducing nerve stimulation. When patients have a positive and good attitude, it would increase their resistance, and would also have a certain role in reducing the spread of cancer cells. To sum up, breast cancer patients need chemotherapy after surgery, which would cause psychological and physiological discomfort. Music relaxation training can reduce the stress response of tumor patients, regulate the emotion of tumor patients, reduce anxiety and depression, improve physical symptoms, alleviate pain, and enhance immune function.

## 4. Positive role of music relaxation training in alleviating the adverse reactions of chemotherapy in breast cancer patients

### 4.1. Alleviating adverse reactions of chemotherapy

The patient has just undergone the treatment of breast cancer surgery and is still in a state of serious psychological and physiological stress. The ongoing chemotherapy would further weaken the immune system of the body, reduce the body’s resistance, and produce various forms of adverse reactions. Playing some gentle music during relaxation training can create a comfortable and calm treatment environment for patients, eliminate the negative impact of physical and mental pressure, restore physical and mental balance and harmony, and help patients better cope with the pain caused by cancer. In this way, the patient’s heart rate, respiration, blood pressure, adrenaline secretion, etc., would become stable, and the adverse reactions during chemotherapy would also be reduced.

### 4.2. Reducing the anxiety of patients during chemotherapy

Anxiety is a complex emotional state of fear of adverse consequences. A certain amount of anxiety helps to improve the psychological tension of the body and enhance the adaptability to stressors. If it is too strong, it would weaken this ability, and if it is too weak, it would lack an objective evaluation of threatening situations. Relaxation training combined with music can make the whole body of the patient enter a relaxed state, and keep the heart, brain and other important organs in a stable state. Anxiety would decrease with the gradual relaxation of the body functions. The mood of the patient would become better, and the anxiety state would become weaker, which is conducive to improving the treatment effect during chemotherapy.

## 5. Methods and techniques of music relaxation training

### 5.1. Music therapy system

**FIGURE 2:**
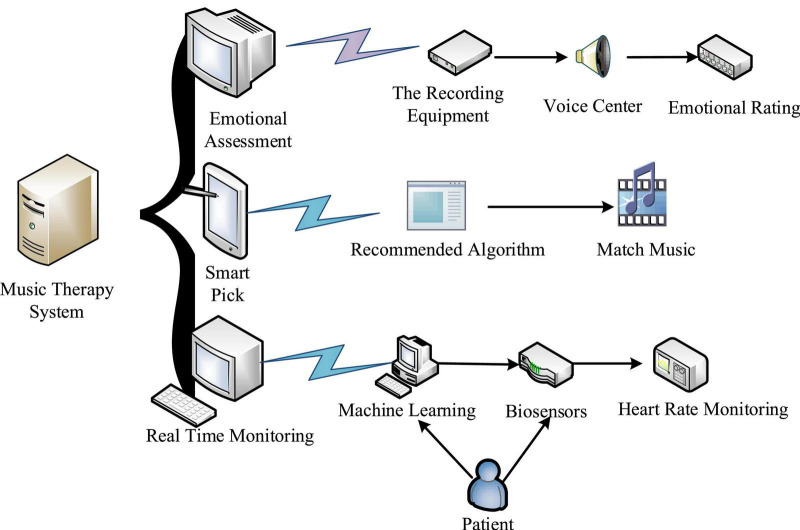
Overall framework of music therapy system.

Combined with the neural network robot system, this manuscript proposes a new music therapy system. As shown in Figure 2, the specific functions of the system are divided into three parts: emotional evaluation, intelligent selection and real-time monitoring.

Affective assessment is the emotional assessment of breast cancer patients in the form of voice. The voice recorder or other intelligent voice equipment is used to collect the patient’s words, and then stored in the voice data center. Finally, the emotional state of the patient is divided into three levels: relaxed, normal, and excited by voice analysis equipment.

#### 5.1.1. Intelligent selection

According to the evaluation results, different types of music are matched for patients with different emotional states through music recommendation algorithms, that is, the first stage of music therapy.

#### 5.1.2. Real time monitoring

This part mainly uses machine learning technology and physiological sensors to monitor the heart rate indicators of breast cancer patients in real time, and then analyzes the current psychological status of patients according to specific indicators. When the patient’s heart rate index changes constantly, the index information would be transmitted to the computer in time, and then the computer would judge the heart rate index in the form of machine learning.

### 5.2. Establishment of music therapy music library

As the emotional state is divided into three levels: relaxed, normal and excited, the music therapy music library is also mainly composed of relaxed, normal and excited music. In addition, digital signal processing technology is used to describe the characteristics of different types of music, quantify the characteristics, and classify the types of music. The music library selects thousands of different styles of music works, and then extracts sound features from their music signals. Special diagnosis extraction includes melody speed, rhythm, frequency, length of time, melody, timbre, lyrics features, etc.

### 5.3. Feedback regulation system of music therapy based on biosensor

Biosensors are sensitive devices to biological reactions, which can convert the concentration of biological reactions into electrical signals (Sopan and Patil, 2022). The biosensor has the advantages of good selectivity, high sensitivity, fast analysis speed and low cost, and it can conduct online continuous monitoring in complex systems. This system uses the biosensor module that can be used for ECG monitoring to collect the heart rate information of breast cancer patients in real time. Heart rate information can reflect the psychological state of patients in real time. When patients are excited, normal and relaxed, their heart rate signals would change to varying degrees. The feedback regulation system would have a set threshold value, and then automatically adjust the music type and level according to the threshold value and heart rate signal value. The specific adjustment mechanism is that when the heart rate signal is greater than the threshold value, the system would play highly soothing music; when the heart rate signal is lower than the threshold value, the system would play gently soothing music to adjust the patient’s emotional state; when the heart rate signal value is between two thresholds, the system would play moderate soothing music.

### 5.4. Music recommendation algorithm for alleviating adverse reactions of chemotherapy in breast cancer patients

**FIGURE 3:**
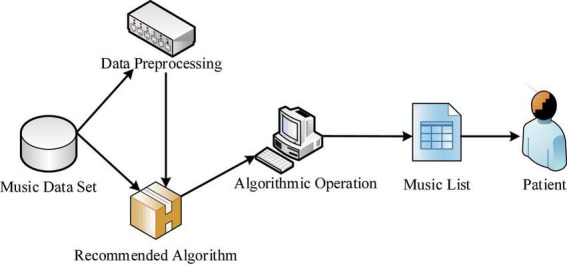
Application process of recommendation algorithm in music recommendation.

In order to make the recommendation effect more comprehensive, the music recommendation algorithm proposed in this manuscript to alleviate the adverse reactions of chemotherapy in breast cancer patients includes content-based recommendation algorithm, collaborative filtering based recommendation algorithm, Item based Collaborative Filtering (Item-CF) recommendation algorithm and recommendation algorithm based on the weighted fusion of content and collaborative filtering. The application process of the recommendation algorithm is shown in Figure 3. First, data preprocessing and recommendation algorithm processing are performed on the music dataset, and then the corresponding song list would be generated after the operation, and finally recommended to breast cancer patients.

#### 5.4.1. Content based recommendation algorithm

According to the text information of music content, the preference matrix of breast cancer patients is obtained by using the Term Frequency - Inverse Document Frequency (TF-IDF) method. Assuming that the set of given music is *O* = {*O*_1_,*O*_2_,*O*_3_,⋯,*O*_*q*_}, the characteristic (key) phrase is *I* = {*x*_1_,*x*_2_,*x*_3_,⋯*x*_*m*_}, and *O*_*y*_ represents the fourth music, the formula of word frequency is:


(1) $$G_{T\,F}\,\left(x,y\right)=\frac{f\,\left(x,y\right)}{\sum_{k\in y}f_{k,y}}$$

Among them, *f*(*x*,*y*) is the number of occurrences of word *x* in music *y*; $\sum_{k\in y}f_{k,y}$ is the total number of occurrences of all words in music *y*; *k* ∈ *y* is the number of occurrences of words in music *y*. The anti document frequency formula is:


(2) $$G_{I\,D\,F}\,\left(x\right)=\log\frac{Q}{q\,\left(x\right)}$$

Among them, *Q* refers to the number of all music, and *q*(*x*) refers to the number of music that has appeared in the feature word *x* of *q*.

The combined TF-IDF weight of feature word *x* in music *y* is calculated as:


(3) $$G_{T\,F-I\,D\,F}\,\left(x,y\right)=G_{T\,F}\,\left(x,y\right)\times G_{I\,D\,F}\,\left(x\right)$$

Among them, *G*_*TF*−*IDF*_(*x*,*y*) represents the word corresponding to the characteristic word *x* in the *y* music. It is normalized:


(4) $$Z_{y\,x}=\frac{G_{T\,F-I\,D\,F}\,\left(x,y\right)}{\sqrt{\sum_{x=1}^{\left|I\right|}G_{\text{TF}-I\,D\,F}\,{\left(x,y\right)}^{2}}}$$

Among them, *Z*_*yx*_ refers to the normalization of the *x* word of the *y* music, so the music preference matrix of breast cancer patients can be obtained:


(5) $$G_{u}=\left(\begin{array}{c} Z_{11}\,\begin{array}{cc} & Z_{12}\,\begin{array}{cc} & \cdots \,\begin{array}{cc} & Z_{1\,q} \end{array} \end{array} \end{array}\\ Z_{21}\,\begin{array}{cc} & Z_{22}\,\begin{array}{cc} & \cdots \,\begin{array}{ccc} & Z_{2\,q} & \end{array} \end{array} \end{array}\\ \vdots \,\begin{array}{ccc} & & \vdots \,\begin{array}{ccc} & & \vdots \end{array} \end{array}\\ Z_{q\,1}\,\begin{array}{cc} & Z_{q\,2}\,\begin{array}{cc} & \cdots \,\begin{array}{ccc} & Z_{q\,q} & \end{array} \end{array} \end{array} \end{array}\right)$$

#### 5.4.2. Collaborative filtering recommendation algorithm

Collaborative filtering algorithm is a more famous and commonly used recommendation algorithm, which is based on the mining of user historical behavior data to find user preferences, and predict the products that users may like to recommend. It must first find “similar breast cancer patients”, and then look for “similar items that breast cancer patients like”. First, cosine similarity is used:


(6) $$Z_{u\,v}=\frac{\left|Q\,\left(u\right)\,⋂Q\,\left(v\right)\right|}{\sqrt{\left|Q\,\left(u\right)\right|\,\left|Q\,\left(v\right)\right|}}$$

Formula (6) is improved, including:


(7) $$Z_{uv}=\frac{\sum_{a\in Q\left(u\right)\cap Q\left(v\right)}\frac{1}{\mathrm{lg}\left(1+\left|Q\left(a\right)\right|\right)}}{\sqrt{\left|Q\left(u\right)\right|\left|Q\left(v\right)\right|}}$$

The reciprocal part of the molecule in Formula (7) is used to punish the popular music in the common preference list of breast cancer patients *u* and *v*, and reduce the impact of popular songs on the similarity of breast cancer patients. The formula of breast cancer patients’ preference for songs is:


(8) $$G\,\left(u,y\right)=\sum_{v\in S\,\left(u,K\right)\,⋂Q\,\left(y\right)}Z_{u\,v}\,R_{v\,y}$$

Among them, *G*(*u*,*y*) refers to the preference degree of breast cancer patients *u* for song *y*; *S*(*u*,*K*) refers to the first *K* breast cancer patients with the most similar interest to breast cancer patients *u*; *Q*(*y*) refers to the collection of breast cancer patients’ behavior history for song *y*; *Z*_*uv*_ refers to the preference similarity between breast cancer patients *u* and *v*; *R*_*vy*_ refers to the preference score matrix of breast cancer patients for song *j*.

#### 5.4.3. Item-CF based recommendation algorithm

Item-CF recommendation algorithm is to find similar items through interest items and recommend similar items to breast cancer patients. The similarity between songs is:


(9) $$Z_{h\,y}=\frac{\left|Q\,\left(h\right)\,⋂Q\,\left(y\right)\right|}{\left|Q\,\left(h\right)\right|}$$

Among them, |*Q*(*h*)| indicates the number of songs *h* loved by multiple breast cancer patients, and the molecule indicates the number of songs *h* and *y* loved by multiple breast cancer patients. After punishing popular music, the similarity is calculated as:


(10) $$Z_{h\,y}=\frac{\left|Q\,\left(h\right)\right|\,⋂\left|Q\,\left(y\right)\right|}{\sqrt{\left|Q\,\left(h\right)\right|\,\left|Q\,\left(y\right)\right|}}$$

Formula (10) reduces the weight of song *y* and reduces the possibility that any song is similar to a popular song.

The Item-CF recommendation algorithm is to first establish an inverted list of breast cancer patients to songs to get the corresponding relationship between breast cancer patients and songs; secondly, a co-occurrence matrix is constructed through the inverted list, and the similarity between the two music is calculated according to Formula (10) to obtain the similarity matrix between each music; finally, the preference of breast cancer patients for songs is calculated. The formula for calculating the preference of breast cancer patients for songs is:


(11) $$G\,\left(u,y\right)=\sum_{y\in S\,\left(h,K\right)\,⋂Q\,\left(u\right)}Z_{h\,y}\,R_{u\,y}$$

Among them, *Q*(*u*) refers to the collection of songs loved by breast cancer patients *u*; *S*(*h*,*K*) refers to the collection of the top *K* songs most similar to song *h*; *Z*_*hy*_ refers to the similarity between music *h* and music *y*; *R*_*uy*_ refers to the preference score of breast cancer patients *u* for music *y*.

D. Music recommendation algorithm based on weighted fusion of content and collaborative filtering.

The algorithm preference formula is:


(12) $$G=\beta \,P_{u}+\left(1-\beta \right)\,P\,\left(u,y\right),\beta \in R,0\leq \beta \leq 1$$

Among them, β refers to the weight of breast cancer patients’ preference matrix, and (1−β) refers to the weight of breast cancer patients’ preference for songs in the collaborative filtering algorithm. The lower the value of β, the greater the impact of similar preferences between breast cancer patients or items on recommendations. With the increase of the value of β, the impact of music content on recommendations is also increasing. The first *K* values are taken, and *K* types of music lists are obtained, which are recommended to corresponding breast cancer patients *u*.

## 6. Evaluation of experimental results of new music therapy in alleviating adverse reactions of chemotherapy in breast cancer patients

**FIGURE 4:**
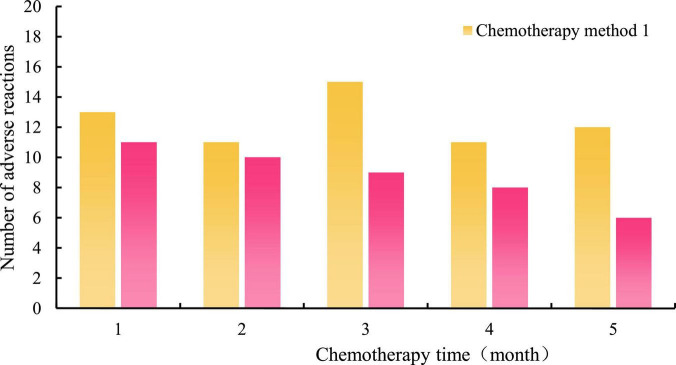
Number of adverse reactions of patients under two chemotherapy methods.

In order to better alleviate the adverse reactions of chemotherapy in breast cancer patients, this manuscript applies the neural network robot system to music training, forming a new music therapy. The following experiments are designed for the practical effect of the new music therapy. This manuscript first investigates the number of adverse reactions of breast cancer patients during chemotherapy in a large tumor hospital under the application of new music therapy. Among them, chemotherapy method 1 represents conventional treatment techniques, and chemotherapy method 2 represents new music therapy. The specific time is divided into 1, 2, 3, 4, and 5 months. The investigation results are shown in Figure 4.

It can be seen from the histogram in Figure 4 that during the 5-month chemotherapy period, the number of adverse reactions of breast cancer patients using chemotherapy method 1 exceeded 10 times per month, and did not decrease gradually with the increase of chemotherapy time. In the application of chemotherapy method 2, although the number of adverse reactions was 10 or more 1 and 2 months later, the number of adverse reactions in the following 3 months was significantly reduced, and the overall trend was gradually reduced with the increase of chemotherapy time.

**FIGURE 5:**
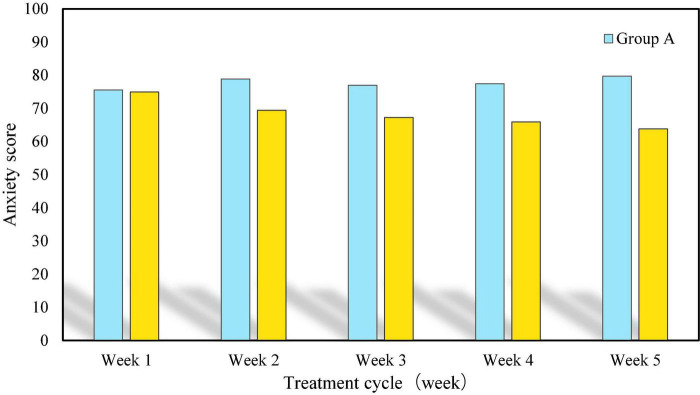
Anxiety score of breast cancer patients within 5 weeks under two treatment methods.

**FIGURE 6:**
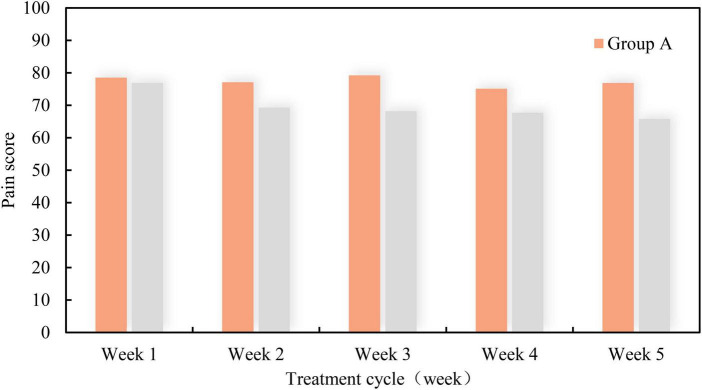
Pain score of breast cancer patients within 5 weeks under two treatment methods.

Anxiety and pain are the most common physical phenomena in breast cancer patients during chemotherapy. If the treatment can reduce the anxiety and pain of patients, then the treatment is undoubtedly successful. In order to test whether the new music therapy can reduce the anxiety and pain of patients, the anxiety scores and pain scores of breast cancer patients within 5 weeks were investigated using the new music therapy and conventional therapy. Among them, patients in Group A applied conventional therapy, while patients in Group B applied new music therapy. The higher the score, the stronger the sense of anxiety and pain. The specific findings are shown in Figures 5, 6.

In the above histogram, the first represents the score of anxiety within 5 weeks, and the second represents the score of pain within 5 weeks. From the weekly score, with the increase of treatment time, the anxiety and pain of patients in Group A did not decrease significantly, and the score basically remained between 75 and 80. In contrast, although the anxiety and pain of patients in Group B were more than 70 points in the first week, they gradually decreased from the second week, and the scores were lower than 70 points. Obviously, with the increase of treatment time, the anxiety and pain of patients in Group B decreased, which indicates that the application of new music therapy is effective.

**FIGURE 7:**
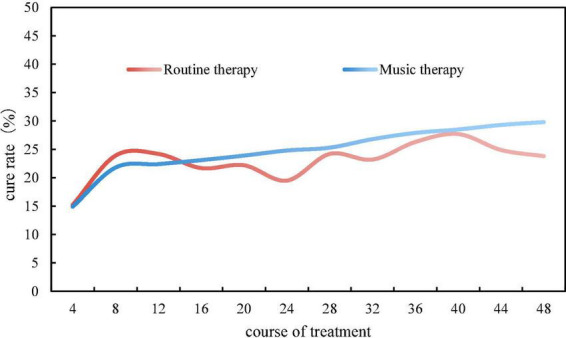
Cure rate under two treatment methods.

As a common malignant tumor, breast cancer also has a certain cure rate. This manuscript proposes that the main purpose of the new music therapy is to alleviate the adverse reactions of chemotherapy in breast cancer patients, but whether it can make a contribution to the cure rate of breast cancer still needs practice. In the case of music therapy and conventional therapy, the cure rate of breast cancer patients in a large tumor hospital within 48 courses of treatment was investigated. Every four courses of treatment were a stage. The investigation results are shown in Figure 7.

It can be concluded from the trend of the curve that the cure rate of breast cancer patients under conventional treatment did not increase gradually with the increase of the course of treatment, and the cure rate at each stage fluctuated greatly, but the overall cure rate was below 25%. The cure rate of breast cancer patients under music therapy was lower than that of conventional therapy in 4, 8, and 12 courses of treatment, because the application of new treatment methods is not very suitable. From the 16th course of treatment, the cure rate showed a steady rise, but the overall cure rate was also below 30%. In contrast, the overall cure rate of music therapy was significantly higher than that of conventional therapy, about 7.84% higher.

It is common for breast cancer patients to have adverse reactions during chemotherapy, and the relevant treatment methods must be able to alleviate them. The above surveys showed the effect of the new music therapy. In order to have a more comprehensive and objective understanding of the practical results of this treatment method, this manuscript investigated the satisfaction of patients, family members, therapists and hospital leaders in a large cancer hospital with the new music therapy. The specific samples were 150 patients, 150 family members, 150 therapists and 150 hospital leaders. The degree of satisfaction was divided into three levels: very satisfied, satisfied and dissatisfied. The survey results are shown in Table 1.

**TABLE 1:** Satisfaction of four categories of personnel with new music therapy.

	Very satisfied		Satisfied		Dissatisfied	
	Number of people	Proportion (%)	Number of people	Proportion (%)	Number of people	Proportion (%)
Patient	89	59.3	43	28.7	18	12
Family members	85	57.7	41	27.3	24	16
Therapist	78	52	47	31.3	25	16.7
Hospital leaders	69	46	52	34.7	29	19.3

According to the data in this table, most of the four types of people were very satisfied with the new music therapy, and very few were dissatisfied, which means that the new music therapy is recognized by everyone.

## 7. Conclusion

Breast cancer is a common malignant tumor disease among many cancers, and many patients are troubled by adverse reactions during chemotherapy. During routine chemotherapy, anxiety and pain have always been the “nightmare” that patients are difficult to get rid of. With the progress of science and technology and the continuous updating of therapeutic techniques, music therapy has gradually entered the field of chemotherapy. In this manuscript, the neural network robot system is applied to music relaxation training, forming a new type of music therapy, and it is used to alleviate the adverse reactions of chemotherapy in breast cancer patients. The research showed that the new music therapy is effective, and it also provided reference value for the development of disease diagnosis and examination technology in the future.

## Data availability statement

The original contributions presented in this study are included in the article/supplementary material, further inquiries can be directed to the corresponding author.

## Author contributions

YT and HY: writing – original draft preparation. YL and HY: editing data curation and supervision. All authors contributed to the article and approved the submitted version.
